# Advances in (Bio)Sensors for Physiological Monitoring: A Special Issue Review

**DOI:** 10.3390/s26020633

**Published:** 2026-01-17

**Authors:** Magnus Falk, Sergey Shleev

**Affiliations:** 1 Department of Biomedical Science, Malmö University, 20506 Malmö, Sweden; 2 Biofilms Research Center for Biointerfaces, Malmö University, 20506 Malmö, Sweden; 3 Citizen Health Research Center, Malmö University, 20506 Malmö, Sweden

**Keywords:** physiological monitoring, wearable (bio)sensors, continuous health monitoring, multimodal sensing, artificial intelligence in biosensing

## Abstract

Physiological monitoring has become an inherently interdisciplinary field, merging advances in engineering, chemistry, biology, medicine, and data analytics to create sensors that continuously track the vital signals of the body. These developments are enabling more personalized and preventive healthcare, as wearable (bio)sensors and intelligent algorithms can detect subtle physiological changes in real-time. In the Special Issue ‘Advances in (Bio)Sensors for Physiological Monitoring’, researchers from diverse domains contributed 18 papers showcasing cutting-edge sensor technologies and applications for health and performance monitoring. In this review, we summarize these contributions by grouping them into logical themes based on their focus: (1) cardiovascular and autonomic monitoring, (2) glucose and metabolic monitoring, (3) wearable sensors for movement and musculoskeletal health, (4) neurophysiological monitoring and brain–computer interfaces, and (5) innovations in sensor technology and methods. This thematic organization highlights the breadth of the research, spanning from fundamental sensor hardware to data-driven analytics, and underscores how modern (bio)sensors are breaking traditional boundaries in healthcare.

## 1. Introduction

**Figure 1:**
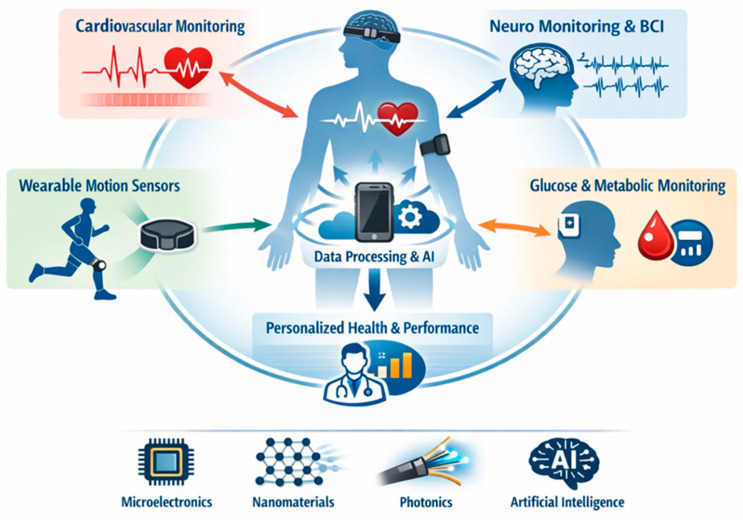
Schematic overview of modern (bio)sensing approaches for physiological monitoring.

Physiological monitoring through (bio)sensing has become a cornerstone of modern healthcare and performance optimization. Advances in microelectronics, nanomaterials, photonics, and artificial intelligence now enable continuous, real-time, and nonintrusive monitoring of both physical signals (e.g., blood pressure, temperature, movement, electrophysiology) and chemical signals (e.g., glucose, metabolites). As we argued in our recent editorial, the notion of a “biosensor” should no longer be limited to devices employing biological recognition elements, but rather should be extended to encompass all sensing technologies that enable physiological monitoring, regardless of their recognition or transduction principle [1]. The Special Issue ‘Advances in (Bio)Sensors for Physiological Monitoring’ captured this broadened vision (Figure 1). The 18 contributions span from commercial continuous glucose monitors to deep-learning-based non-invasive metabolic sensing, from fNIRS hyperscanning in social interactions to ultra-low-power neural amplifiers. This diversity reflects a rapidly converging field where sensing, computation, and physiology intersect and aligns with the broader shift in wearable electronics toward integrated, multimodal systems that combine sensing, computation, and connectivity to support continuous and personalized healthcare, as highlighted in recent field-level perspectives [2].

This interdisciplinarity mirrors broader trends across the biosensing landscape. In the autonomic–cognition interface, systematic reviews have demonstrated that indices of autonomic regulation, particularly heart rate variability (HRV), are robustly associated with cognitive and affective functioning across health and disease [3,4]. In metabolic sensing, recent research has highlighted the rapid evolution of non-invasive and minimally invasive approaches, including optical, electrochemical, and data-driven modalities [5,6,7]. Likewise, overviews of wearable biosensors have emphasized how advances in microelectronics, nanomaterials, and machine learning are transforming physiological monitoring into an integrated and personalized healthcare paradigm [8,9]. In movement and musculoskeletal health, surveys of wearable technologies in sports and rehabilitation have illustrated how inertial, optical, and electromyographic sensing systems are transitioning from controlled laboratory validation to continuous real-world use [10,11]. Together, these perspectives frame the contributions of this Special Issue as representative of the broader evolution of physiological monitoring by bridging fundamental sensor design, advanced analytics, and applied health science. Building on this framing, this review organizes the contributions into five major themes:

1. Cardiovascular and autonomic monitoring.
2. Glucose and metabolic sensing.
3. Wearable sensors for movement and musculoskeletal health.
4. Neurophysiological monitoring and brain–computer interfaces.
5. Innovations in sensor technology and methods.

Each section introduces the relevant contributions from the Special Issue and situates them within the context of select external works. This dual perspective not only emphasizes the novelty of the reported studies, but also how they advance broader trajectories in (bio)sensing for health and performance monitoring.

## 2. (Bio)Sensors for Physiological Monitoring

### 2.1. Cardiovascular and Autonomic Responses in Health Monitoring

Several studies have examined how continuous cardiovascular and autonomic measurements can predict health outcomes and performance. Xue and Romero-Ortuno presented two complementary works using continuous blood pressure, heart rate, and near-infrared spectroscopy (NIRS) signals during an active stand test (standing up from lying down) in older adults [12,13]. In one study on a large cohort from the Irish Longitudinal Study on Ageing, they found that unprocessed (“raw”) physiological signals carried more predictive information for adverse outcomes than conventionally pre-processed signals. In particular, raw features like continuous systolic/diastolic blood pressure and frontal lobe oxygenated hemoglobin (O_2_Hb) changes captured orthostatic intolerance and even 4-year mortality risk more effectively, whereas pre-processed summaries missed some of these sustained responses. These findings underscore the value of high-resolution, raw signal monitoring for risk stratification (e.g., detecting orthostatic hypotension early) and suggest that retaining rich signal data can improve predictive modeling for clinical outcomes. In a related pilot study focusing on older women, Xue and Romero-Ortuno applied functional data analysis (FDA) to the continuous stand-test signals. This approach revealed subtle but significant group differences: for example, women under 70 showed faster and larger heart rate responses after standing than those over 70, and those with initial orthostatic hypotension had distinct blood pressure drop patterns around 10 s post-stand. Similarly, overweight participants (BMI ≥ 25) exhibited altered cerebral hemodynamics (lower NIRS-measured deoxyhemoglobin and O_2_Hb levels at specific intervals) compared to normal-weight participants. Notably, no differences were seen by antihypertensive medication use or frailty status, suggesting that age and vascular factors were the primary drivers of response variation. Together, these studies highlight how continuous cardiovascular and neurovascular monitoring during a physiological challenge can unmask age-related or health-related impairments that average measures overlook, reinforcing the importance of dynamic autonomic tests in geriatric health assessments.

**Figure 2:**
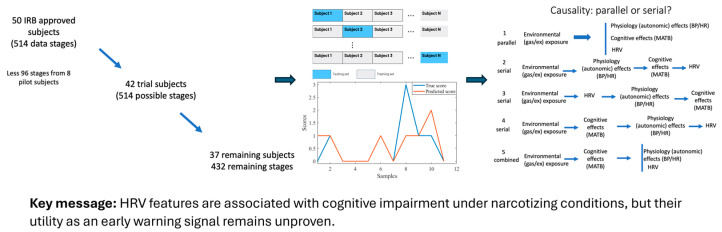
Editorial synthesis of the study by Freiberger et al., which examined the association between heart rate variability (HRV) and cognitive performance impairment in divers under hyperbaric and narcotizing conditions.

Expanding the context to extreme environments, Freiberger et al. investigated whether heart rate variability (HRV), a metric of autonomic nervous system activity, could serve as an early warning of cognitive impairment in divers breathing narcotic gas mixtures [14]. In a series of simulated dives with a cognitive performance task, they analyzed 23 HRV features under both normal conditions and conditions inducing nitrogen narcosis or high CO_2_/O_2_ exposure. The study found that certain HRV metrics significantly correlated with decrements in cognitive performance during deep dives. In particular, reductions in high-frequency HRV power (HF_nu_, reflecting parasympathetic activity) and a lower count of normal-to-normal beat interval variations (NN50 count) were associated with the impaired performance of memory, attention, and motor tasks under narcosis. Additional HRV parameters (e.g., RMSSD, Poincaré plot width) also showed associations with performance when stratifying resting vs. exercise conditions. These results suggest that autonomic changes measured via HRV could serve as physiological biomarkers of impending cognitive deficits in divers, potentially offering a non-intrusive safety monitor for high-risk underwater operations (Figure 2). Overall, the works by Xue and Romero-Ortuno and Freiberger et al. demonstrate how continuous cardiovascular/autonomic sensing, whether in clinical orthostatic tests or in operational settings, can yield actionable insights, from identifying older individuals at fall risk to alerting divers to cognitive risk under extreme conditions.

Together, these studies resonate with a growing body of evidence that positions autonomic and cardiovascular metrics as integrative indicators of both physiological and cognitive states. Forte et al. (2019) demonstrated in a systematic review that HRV is robustly linked to executive and memory functions, underscoring its role as a marker of neurovisceral integration [3]. More recently, Nicolini et al. (2024) provided longitudinal evidence that HRV predicts future cognitive performance in adults, reinforcing the idea that cardiac autonomic regulation reflects broader adaptive capacity across domains of health [4]. Recent empirical studies further support this link, showing that reduced HRV is associated with cognitive impairment and poorer cognitive performance, as well as with longitudinal cognitive decline from midlife into older age [15,16]. Beyond cognition, HRV and related measures have been associated with stress resilience, sleep quality, and metabolic efficiency, making them valuable targets for preventive monitoring and personalized interventions [17,18]. Elevated autonomic flexibility, reflected in higher HRV, has been linked to improved emotional regulation and adaptive recovery from stress, whereas chronically reduced variability often signals cumulative physiological strain. As wearable sensors now enable long-term HRV tracking in everyday life, these metrics are increasingly being used to quantify allostatic load and guide individualized lifestyle or training adjustments, contributing to emerging frameworks that conceptualize a digital phenotype of allostatic load derived from continuous, multimodal physiological monitoring [19].

### 2.2. Glucose Monitoring and Metabolic Sensors

Metabolic sensing represents one of the most active and clinically impactful domains of wearable (bio)sensor research. Glucose monitoring, in particular, serves as a cornerstone for diabetes management and is increasingly being explored for preventive health, fitness, and nutrition applications. Continuous glucose monitors (CGMs) exemplify how biochemical sensing has moved from clinical to everyday use, providing real-time insights into metabolic responses to diet, exercise, and stress. Yet, despite remarkable technological maturity, important challenges remain especially regarding accuracy, calibration, and usability in non-diabetic populations.

**Figure 3:**
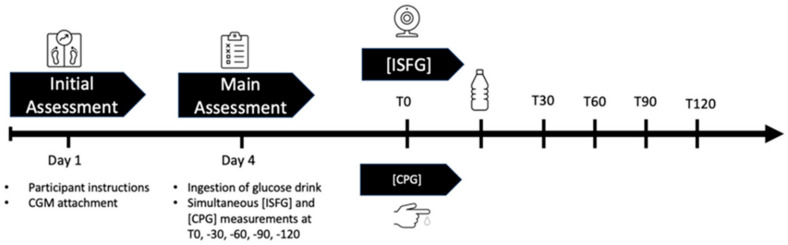
Graphical overview of the study design: On day 1 (initial assessment), participants were instructed and CGM sensors were applied onto their attachment site. On day 4, after a carbohydrate-rich nutrition for the last three days and an 8–12 h fast, they were asked to ingest a glucose drink to start the OGTT. Simultaneous ISFG and CPG were taken at T0, T30, T60, T90, and T120. Abbreviations: CGM = continuous glucose measurement, ISFG = interstitial fluid glucose, CPG = capillary glucose, OGTT = oral glucose tolerance test, T0 = fasting baseline, T30, T60, T90, T120 = 30, 60, 90, 120 min post-OGTT.

Monitoring biochemical signals is another key theme, illustrated by advances in glucose sensing for metabolic health. Fellinger et al. evaluated the performance of a popular continuous glucose monitor (CGM), the FreeStyle Libre 2, in healthy young adults without diabetes [20]. While CGMs are widely used for managing diabetes, their accuracy in people with normal glucose regulation (e.g., for fitness or diet tracking) had been less clear. In a controlled oral glucose tolerance test involving 44 healthy males, the interstitial glucose readings from the wearable sensor were compared against reference blood glucose at several time points (Figure 3). The CGM showed a mean absolute relative difference (MARD) of ~12.9% from the reference, and 100% of its readings fell within the clinically acceptable error zones (A and B) of the consensus error grid. This indicates that the sensor is generally reliable for tracking glucose trends in non-diabetic contexts. However, the authors found a consistent bias: the CGM systematically under-reported glucose levels relative to finger-prick measurements at all time points (by about 5–10 mg/dL on average, *p* < 0.001). In other words, after a glucose challenge, the sensor readings were significantly lower than plasma values, likely due to the well-known lag of interstitial glucose sensing. These results suggest that wearable CGMs can be useful for healthy individuals by providing convenient continuous monitoring, but their absolute values should be interpreted with caution in clinical decision-making for non-diabetic use cases. The work provides a valuable benchmark of the accuracy of CGMs in a healthy population and highlights the need for context-aware calibration when such sensors are used beyond diabetic settings.

While Fellinger et al. examined a commercial sensor, the contribution by Chellamani et al. focused on developing a new non-invasive glucose monitoring approach using photoplethysmography (PPG) signals and deep learning. They proposed a Deep Sparse Capsule Network (DSCNet) model that analyzes the subtle features in the pulse waveform (PPG) to estimate blood glucose levels without finger sticks [21]. The key innovations of their approach include advanced signal preprocessing (Savitzky–Golay filtering and moving averages to denoise the PPG) and an attention mechanism that helps the model focus on relevant pulse characteristics while ignoring noise. By extracting both time-domain and spectral features from PPG and feeding them into the capsule network, their system achieved high accuracy in glucose prediction across diverse subjects. This study reflects a broader trend of leveraging AI to enhance biosensors: rather than measuring glucose directly (as electrochemical CGMs do), it infers glucose from patterns in a simpler optical sensor, potentially lowering the cost and increasing comfort. Such non-invasive techniques, if validated further, could greatly improve patient compliance and enable continuous metabolic monitoring for preventive health.

These contributions align with a broader wave of research advancing metabolic sensing toward more accessible, continuous, and intelligent health monitoring. Min et al. (2025) reviewed minimally and non-invasive glucose monitoring technologies, outlining the progress in optical, electromagnetic, and wearable modalities that aim to reduce reliance on invasive sampling [5]. Complementing this, Gao et al. (2023) summarized the developments in wearable and flexible electrochemical sensors for sweat analysis, demonstrating how real-time biochemical monitoring can capture metabolic responses to stress, exercise, and diet [22]. Meanwhile, Chan et al. (2024) conducted a scoping review of artificial intelligence-based non-invasive glucose monitoring, highlighting how data-driven models increasingly enable indirect estimation of glucose from optical or physiological signals [23]. Collectively, these reviews point to a dual trajectory: the refinement of established CG monitoring systems alongside the emergence of non-invasive, AI-enhanced platforms for preventive metabolic health. Recent studies have further demonstrated deep-learning-based glucose inference from photoplethysmography signals and emerging electromagnetic sensing strategies that extend beyond optical modalities [24,25]. This convergence signals a shift from reactive diabetes management toward proactive metabolic wellness, where multimodal biochemical sensing informs personalized nutrition, physical activity, and lifestyle interventions.

### 2.3. Wearable Sensors for Movement and Musculoskeletal Health

Monitoring movement and musculoskeletal function has become a key frontier in wearable sensing. Accurate, continuous assessment of posture, motion dynamics, and physical strain can not only inform athletic performance optimization, but also rehabilitation, ergonomics, and the prevention of work-related musculoskeletal disorders. Traditional motion capture systems, though precise, remain confined to laboratory settings; in contrast, wearable inertial, optical, and textile-based sensors now allow for movement to be quantified in real-world environments.

**Figure 4:**
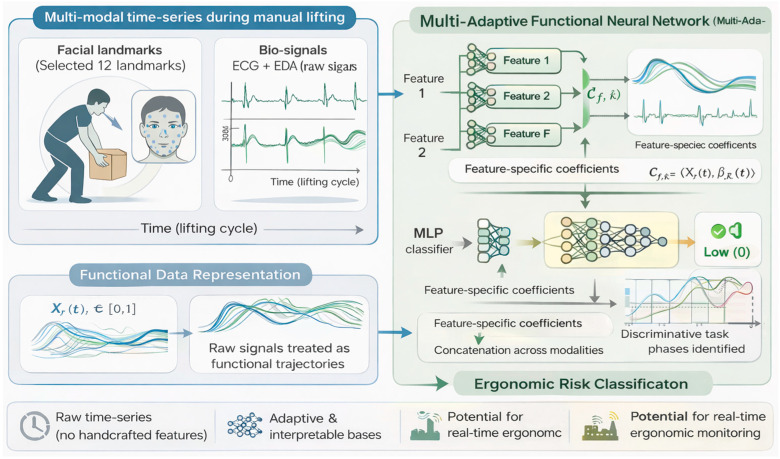
Conceptual overview of the multimodal sensing and Multi-Adaptive Functional Neural Network (Multi-AdaFNN) framework for ergonomic risk classification.

Within the Special Issue, several papers exemplify these developments. Kim et al. tackled the challenge of ergonomic risk classification during manual labor using a multi-modal sensing approach [26]. They collected time-series data from wearable bio-signals (electrocardiography and electrodermal activity) along with video-based facial landmarks as workers performed lifting tasks, aiming to automatically classify the lifting into low-, medium-, or high-injury-risk categories. To analyze this complex data, the authors developed a novel Multi-Adaptive Functional Neural Network that integrated functional data analysis with deep learning. A key feature of the model is its adaptive basis layer that learns personalized temporal features from each sensor stream before fusing them, which helps in handling the different time scales and noise characteristics of physiological vs. video data. The results were promising: by combining facial expression cues with physiological signals, the model achieved the highest accuracy in classifying lift risk, outperforming models that used either type of data alone. The Multi-Adaptive Functional Neural Network not only improved accuracy, but also provided interpretability, where the learned functional basis functions corresponded to distinct phases of the lift (e.g., initial strain visible in facial tension followed by sustained heart rate elevation), highlighting when during the lift the model found risk-indicating patterns. This approach demonstrates how fusing multimodal wearable sensors with AI can lead to more robust occupational health tools, potentially enabling real-time feedback to workers to prevent back injuries (Figure 4).

Closely related to ergonomic monitoring is the use of sensors in analyzing gait and movement patterns for musculoskeletal health. Pascaud et al. explored this in the context of low-back pain (LBP), which often lacks clear biomechanical markers between painful episodes [27]. They studied 34 young adults (some with a history of recurrent LBP, others without) during walking, using motion capture and wearable sensors to quantify trunk and pelvic motion and muscle activity. The focus was on inter-girdle coordination, which is essentially how the rotations of the pelvic girdle and shoulder girdle are coupled during gait. Remarkably, even when patients with LBP were pain-free during testing and had normal gait speed and range of motion, their coordination pattern differed from controls. The LBP group showed a more in-phase (less out-of-phase) relationship between pelvic and thoracic rotations, indicating a stiffer, en-bloc movement of the trunk. No significant differences were found in the conventional gait parameters or muscle activation, suggesting that inter-girdle coordination is a more sensitive marker. The authors argue that this coordinative pattern reflects an adaptive strategy to stabilize the trunk, which might protect against acute pain recurrences, but also signals an underlying vulnerability. These findings point to coordination metrics as a preventative target: interventions (such as specific core exercises focusing on transverse plane mobility) could aim to improve the relative timing between pelvic and trunk motion. Overall, the work illustrates the power of wearable kinematic sensors to detect subtle functional deficits in seemingly healthy individuals, which could be used to personalize rehabilitation and prevent LBP flare-ups. It also complements the theme of ergonomic sensing: both Kim et al. and Pascaud et al. show that sensor-based analyses of movement—whether lifting or walking—can reveal patterns linked to injury risk, enabling proactive management.

Hand function assessment represents another important application of wearable and optical sensing in musculoskeletal health. Vieira et al. evaluated two sensor-based systems for quantifying the range of motion of finger joints—a nine-axis IMU-based glove (accelerometer, gyroscope, and magnetometer) and an infrared camera system—comparing their repeatability and accuracy against traditional goniometry [28]. Using a silicone hand model and a healthy volunteer, the authors showed that both systems exhibited excellent repeatability, with standard deviations being consistently below clinically acceptable thresholds. However, agreement with the goniometer was limited, with a substantial proportion of measurements deviating by more than five degrees. Notably, the two sensor-based systems were more consistent with each other than with the goniometer, suggesting that conventional manual tools may themselves impose significant measurement uncertainty. The study highlights how automated, sensor-based hand assessments can improve objectivity, efficiency, and reproducibility in rehabilitation settings, while also underscoring the need to reconsider traditional reference standards for fine motor evaluation.

These findings extend the vision articulated by Düking et al. (2018), who argued that wearable technologies should integrate biomechanics, mobile applications, and diagnostics to individualize physical activity [10]. The papers in the Special Issue exemplify how such integration is now materializing in ergonomics and gait analysis, offering sensitive markers for injury prevention and rehabilitation. This evolution mirrors the broader trends highlighted by recent reviews, which document how wearable inertial and multi-sensor systems are transitioning from laboratory validation to continuous real-world monitoring of human movement, while also underscoring persistent challenges in task generalization, external load estimation, and dataset bias when translating ergonomic classification models beyond controlled laboratory settings [11,29,30]. Complementing these reviews, Caramaschi et al. (2024) demonstrated how smartphone-based sensing can quantify gait parameters such as walking distance in outdoor environments, emphasizing the critical influence of data quality on measurement accuracy [31]. Such work underscores that, as wearable and mobile sensors move into free-living conditions, signal reliability and calibration become as important as algorithmic sophistication. In this context, wearable motion sensing is advancing from performance tracking toward digital musculoskeletal health monitoring, supporting precision rehabilitation, workplace safety, and personalized training.

### 2.4. Neurophysiological Monitoring and Brain–Computer Interfaces

Monitoring brain activity through non-invasive sensing technologies is rapidly transforming both neuroscience research and clinical practice. Advances in wearable neurotechnologies, particularly in functional near-infrared spectroscopy (fNIRS), electroencephalography (EEG), and hybrid systems, enable brain monitoring in naturalistic settings that were once accessible only through laboratory equipment. These developments open new opportunities for social neuroscience, rehabilitation, and assistive interfaces, bridging the gap between fundamental brain research and practical applications.

**Figure 5:**
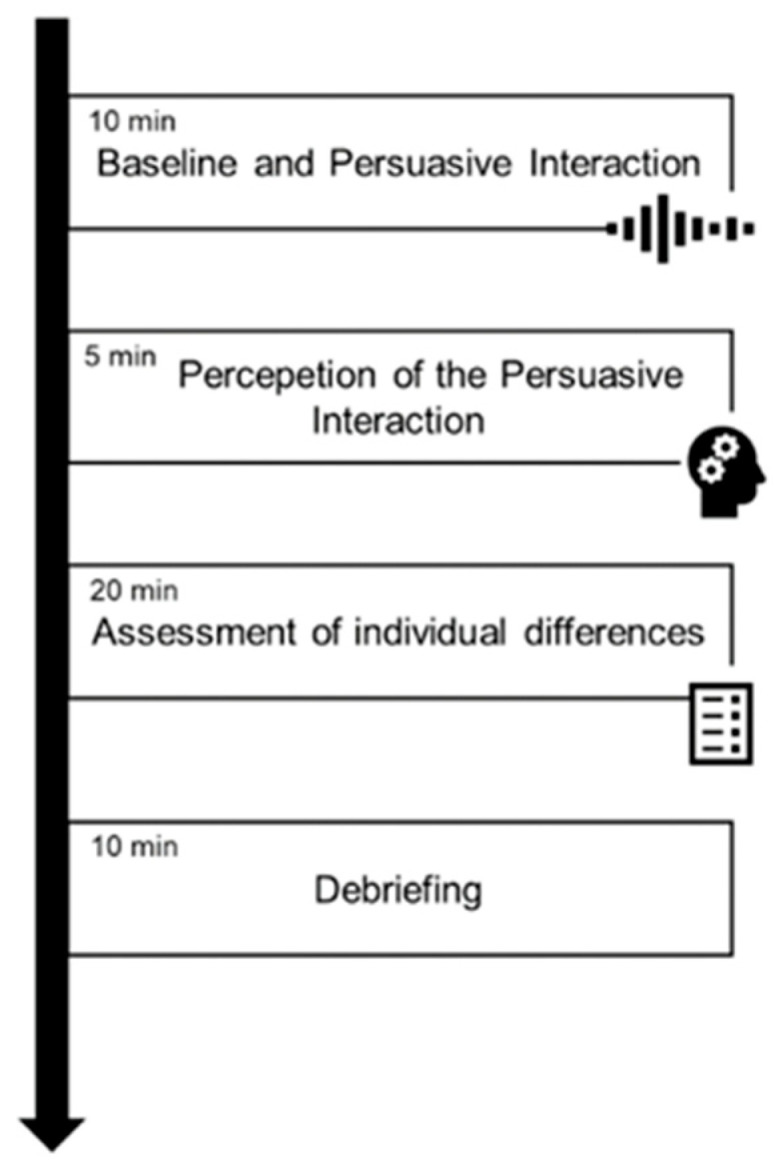
Schematic overview of the experimental procedure. The protocol consisted of four consecutive phases: (i) baseline recording and persuasive interaction (10 min), (ii) perception of the persuasive interaction (5 min), (iii) assessment of individual differences (20 min), and (iv) debriefing (10 min).

Advances in sensors for monitoring brain activity, particularly using non-invasive optical methods, feature prominently in the Special Issue. One innovative application is in social neuroscience: Balconi et al. used fNIRS to monitor brain hemodynamics in pairs of people (dyads) engaged in a persuasion and decision-making task [32]. This hyperscanning approach (simultaneous monitoring of two brains) examined how the prefrontal cortex activity of a “persuader” and a “persuaded” person become synchronized or differentiated over the course of an interaction. The authors introduced a measure of hemodynamic coherence between the two individuals, computed via the similarity (Euclidean distance) of their fNIRS signals over time (Figure 5). They found that during the response phase of the interaction when the second person either agrees or disagrees with the first, the coherence actually decreased (particularly in deoxygenated hemoglobin signals), indicating a divergence in brain activity between the pair. Interestingly, this phase of increased neural dissimilarity does not imply a breakdown of communication, but rather reflects the natural engagement of different cognitive processes as the listener formulates their own stance. These results highlight that brain-to-brain synchrony in a social context can fluctuate with the dynamics of the conversation. More broadly, the study demonstrates the feasibility of using wearable optical sensors (fNIRS headbands) to monitor interactive brain function in real-world tasks. Such hyperscanning methods could have applications in measuring team dynamics, therapy (e.g., patient-therapist interaction), or education by revealing moments of high or low neural coupling between individuals. Balconi et al. also underscore fNIRS’s potential as a practical neuromonitoring tool in live social settings, since it is safe, non-invasive, and relatively tolerant to motion.

While fNIRS provides a window into brain function, extracting maximal information from its signals is an ongoing challenge due to the modality’s slow hemodynamic response and low sampling rate. Han et al. addressed this with a new decoding method called TopoTempNet to improve fNIRS-based brain–computer interfaces (BCIs) [33]. BCIs translate brain signals into commands (often for assistive devices), and fNIRS is emerging as an attractive BCI signal source because it is portable and user-friendly, though it lags in temporal resolution compared to EEG. TopoTempNet tackles this by combining graph theory and deep neural networks to capture both spatial and temporal patterns in fNIRS data. The model constructs multi-level graph features that represent functional connectivity between different brain regions (channels) and feeds these into a hybrid attention-augmented recurrent network (incorporating Transformer and bi-directional LSTM layers). This design allows for the network to weigh the most informative connections and time points for distinguishing mental states. When evaluated on motor imagery tasks from three public datasets, TopoTempNet achieved excellent performance—classification accuracies up to ~90% (with correspondingly high kappa scores) in discriminating different imagined movements. This substantially outperformed prior state-of-the-art fNIRS BCI models. Moreover, by analyzing the learned graph attention weights, the authors showed that the network emphasized specific brain connection patterns that aligned with the known neurophysiological pathways activated during motor imagery. Such interpretability is important for user trust and for refining BCI paradigms. In summary, TopoTempNet demonstrates a powerful approach to overcoming fNIRS’s limitations by fusing connectivity analytics with deep learning, paving the way for more accurate and robust fNIRS-driven BCIs for patients with motor impairments. Its success also reflects a broader theme of the Special Issue: the integration of AI techniques to enhance signal decoding and make sense of complex biosignals in real-time.

Together, these studies align with the broader trajectory of fNIRS and optical neurotechnologies, which is moving from laboratory paradigms toward ecological, social, and AI-augmented applications. Recent work on fNIRS hyperscanning has mapped interpersonal neural synchrony in interactive and clinical settings, showing that coupling between brains varies with task demands and relationship contexts [34,35]. In parallel, BCI research is increasingly leveraging graph-based and deep learning models to capture the spatio-temporal structure of fNIRS and hybrid EEG–fNIRS signals, markedly improving motor-imagery decoding in realistic tasks [36,37,38]. Seen in this light, the Special Issue contributions mirror the field’s shift toward context-aware neurophysiological monitoring, a trajectory further exemplified by recent demonstrations of wireless ear-EEG systems capable of continuous, unobtrusive monitoring of brain states such as drowsiness in real-world settings [39]. Collectively, these developments span applications ranging from social neuroscience use cases (hyperscanning during interaction) to advanced decoding pipelines for assistive brain–computer interfaces.

### 2.5. Innovations in Sensor Technology and Methods

Beyond specific application domains, a number of papers in this Special Issue report fundamental innovations in sensor design, materials, and system integration that collectively expand the capabilities of (bio)sensing technologies. Progress in this area underpins nearly every advance in physiological monitoring, determining which signals can be detected, with what fidelity, and in which environments. Emerging frontiers include miniaturized analog front-ends, nanomaterial-based electrodes, wireless communication systems, and bio-inspired photonic sensing, all converging to make sensing more precise, energy-efficient, and adaptable to real-world conditions.

**Figure 6:**
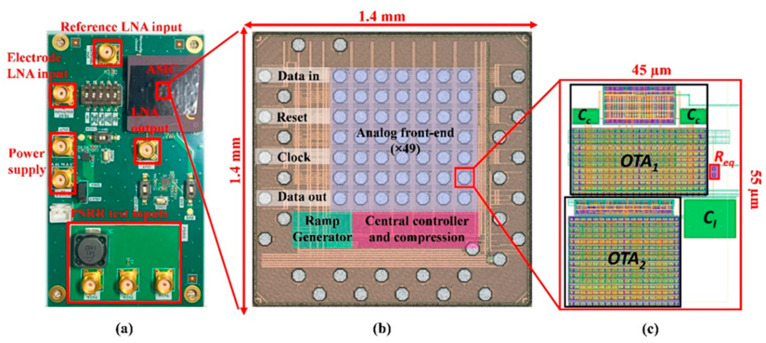
(**a**) Test PCB for analog front-end measurement. (**b**) Micrograph of the ASIC featuring 49 recording channels. (**c**) The layout of the proposed LNA was designed using 28 nm CMOS technology.

A prime example is the development of new sensor hardware for neural monitoring. Ranjbar Koleibi et al. described an ultra-low-power high-performance biopotential amplifier ASIC designed for large-scale neural recording systems [40]. Implemented in a cutting-edge 28 nm CMOS process, their amplifier achieved an exceptionally high input impedance of ~10^11^ Ω (to avoid loading neural electrodes) while maintaining low noise (~11 µV_rms_) and a sizable bandwidth (~7 kHz) suitable for action potentials. Notably, the design eliminates bulky coupling capacitors by using an active low-pass filter for DC suppression, which, combined with the advanced fabrication node, shrank the core area to only 0.0025 mm^2^. It also consumes only 3.4 µW of power, making it feasible to integrate hundreds of channels on implantable chips without thermal or battery concerns. These specs (58 dB gain, NEF ≈ 8.4, PEF ≈ 85) represent a leap forward in the miniaturization and energy-efficiency of neural amplifiers (Figure 6). Such technology could enable next-generation brain implants or wearable EEG devices that record using many more electrodes with high fidelity, advancing both neuroscience research and clinical neuroprosthetics.

Innovation in the materials used for wearables is another important direction. Medina and Child provided a comprehensive review of carbon-based nanocomposite electrodes for non-invasive EEG [41]. They surveyed how mixing carbon nanomaterials (like carbon nanotubes, graphene, carbon black) into soft polymers can produce flexible “dry” electrodes that adhere to the skin and conduct bioelectric signals without the need for gels. These nanocomposite electrodes offer attractive properties for EEG: high conductivity and low contact impedance (thanks to the conductive filler), combined with mechanical compliance and comfort on the scalp. The review discussed various design configurations (e.g., different nanoparticle types, concentrations, and surface patterns) and fabrication techniques (printing, molding, etc.), along with performance comparisons to standard wet electrodes. Many studies show that such electrodes can achieve signal quality comparable to wet electrodes for EEG rhythms while being far more practical for wearable use (no skin prep or gel needed). However, Medina and Child also emphasize challenges that remain, for instance, consistency in manufacturing, long-term stability on hair-bearing skin, and variation in performance metrics across studies. They called for standardized testing protocols for new EEG electrode materials to enable fair benchmarking and accelerate translation of the best designs to market. This aligns with the general message that, as novel biosensor materials proliferate, the community needs common evaluation standards (in terms of impedance, noise, signal-to-noise ratio in actual recordings, etc.) to identify which innovations truly improve upon the status quo. Overall, the review highlights that carbon-based soft electrodes are a promising route to a truly wearable EEG and showcases the intersection of nanomaterials science with sensor development.

Long-term wearability and self-applicability remain major challenges for mobile EEG systems. Addressing this, da Silva Souto et al. evaluated flex-printed electrode grids with pre-applied conductive materials, comparing a curing hydrogel with a novel silicone-based dry material during five- to six-hour mobile EEG recordings [42]. Signal quality was assessed using continuous impedance monitoring and auditory-evoked potentials (AEPs) recorded in morning and afternoon sessions. Both materials enabled reliable EEG acquisition with comparable AEP morphology and signal-to-noise ratios, demonstrating feasibility for prolonged use. However, the impedance dynamics differed: hydrogel electrodes showed a slight decrease in impedance over time, while silicone electrodes exhibited a gradual increase and a higher rate of high-impedance outliers. Despite this, both materials supported high-quality evoked responses and improved wearing comfort relative to conventional gel-based electrodes. The study highlights how material choice critically shapes long-term signal stability, usability, and comfort, and underscores the importance of moving beyond short laboratory recordings toward realistic, day-long EEG monitoring scenarios.

Innovations were also reported in the realm of wireless communication for sensors. Domingues et al. addressed a bottleneck for implantable and wearable devices: how to transmit high-throughput physiological data without wires or large batteries [43]. They designed an optical wireless communication system (OWCS) for implanted neural probes in small animals as an alternative to radio-frequency telemetry. The OWCS uses an infrared LED inside the implant to send data as light through bodily tissue, which is then picked up by an external photodiode receiver. A creative aspect of their setup is a tracking system using a piezoelectric force-sensing floor: as the animal moves in its cage, the external optical receiver adjusts to stay aligned with the implant’s transmitter. In tests with tissue-mimicking phantoms, the team demonstrated a data rate of 5 Mbit/s at 850 nm wavelength through 10 mm of tissue, requiring only about 55 mW of optical power. This exceeds what typical in-body RF devices can do (they often max out around 1–2 Mbit/s due to absorption by tissue) and validates the potential of near-IR light for high-bandwidth implants. The work points toward future implantable sensors that could stream raw biosignals (like neural data or high-res biosensor readings) in real-time without tethering, a significant step for free-moving animal studies and eventually human medical implants. The use of an optical link also avoids the strict frequency regulation and antenna size issues of RF, although it introduces the need for line-of-sight alignment (hence the adaptive tracking solution demonstrated). This OWCS approach, with further development, could broadly impact wearable health monitors as well, for instance, enabling high-data-rate communication between on-body patches and smartphones using safe optical wavelengths.

In the area of lab-on-a-chip diagnostics, Garcia-Torales et al. presented a novel sensor-integrated platform for DNA amplification [44]. They developed a thermal bed for Loop-Mediated Isothermal Amplification (LAMP) assays built using PCB technology for precise temperature control. LAMP is a technique for amplifying DNA at a constant temperature (used in rapid pathogen tests, etc.), but it requires very stable heating. The team’s solution was to design a PCB with copper traces that act as resistive heaters, combined with optoelectronic sensors for feedback, all governed by a PID controller on a microcontroller. The resulting device could maintain the target temperature with ±1 °C accuracy and quickly ramp to temperature in about 2.5 s. FEM simulations confirmed a uniform temperature distribution across the reaction area. This level of control is impressive for a simple, low-cost platform and is crucial for reliable µLAMP (microfluidic LAMP) diagnostics. Additionally, the system monitored the amplification progress via optical means (possibly detecting colorimetric changes in the LAMP reaction). The modular, repairable design on PCB makes it user-friendly and scalable. Such an invention could be the basis of portable genetic testing devices for point-of-care diagnostics, where maintaining exact temperatures in a small, wearable form factor is often challenging. By leveraging inexpensive PCB fabrication, the authors illustrate an accessible path to bring molecular diagnostics into everyday settings (e.g., wearables for health monitoring or environmental testing kits).

Optical and photonic sensor research in the Special Issue was not limited to biological sensing per se; it also drew bioinspiration for engineering applications. Li et al. contributed a comprehensive review of imaging polarimeter designs inspired by animal navigation [45]. While not directly a physiological monitor, this review fits the theme of bio-inspired sensing: many animals (like certain insects) navigate by sensing the sky’s polarization pattern. The authors discussed different structural types of polarization detection units used in imaging polarimeters, categorizing them by how they modulate and split incoming light: time-division multiplexing, spatial (channel) division with subtypes using single-sensor vs. multi-sensor arrangements, and division of focal plane approaches. By analyzing recent advances in these designs (many of which mimic the compound eyes of insects or the polarization sensitivity of marine animals), the review highlights how multi-modal sensor structures can capture polarization information alongside conventional intensity images. Such polarization imaging systems have emerging applications in navigation and positioning (e.g., robotic guidance using skylight cues) and even in biomedical imaging for enhanced contrast. The detailed discussion by Li et al. aims to clarify the sometimes confusing terminology used in prior classifications and provides inspiration for engineers looking to implement polarization sensing by outlining the trade-offs of each structural approach. Including this topic in the Special Issue underlines the breadth of “(bio)sensing”, extending the concept beyond direct health monitoring to also encompass sensors that draw inspiration from biological systems.

Lastly, in the realm of biomechanical sensors, Bouffandeau et al. introduced an impact-based analysis method (IBAM) to assess the mechanical properties of soft tissues, as a non-invasive alternative to palpation [46]. They designed a simple instrumented hammer that delivers a gentle impact to the tissue (or a tissue-mimicking phantom), and from the force–time signal, they extract an indicator (Δt) related to the tissue’s stiffness. In tests on agar phantoms of various stiffness and layer configurations, the IBAM’s stiffness measurements correlated well with those from a standard Dynamic Mechanical Analyzer (DMA) and were more sensitive to stiffness changes than a commercial digital palpation device (MyotonPro). In fact, IBAM could detect smaller differences in Young’s modulus that MyotonPro missed, although its spatial resolution was lower (since the hammer’s impact influences a larger tissue volume). The authors note that while MyotonPro has better localized resolution, the IBAM’s greater sensitivity to stiffness suggests that it could be valuable for screening skin or tissue conditions that alter biomechanical properties (scleroderma, fibrosis, tumors, etc.). The simplicity of the setup—essentially a lightweight hammer and a force sensor—means that it could be made wearable or at least portable, providing quantitative palpation data for clinicians or even being adapted into smart prosthetics or gloves. This study is a good example of how combining a basic sensor (force transducer) with clever signal analysis can yield a new biomedical tool. It “paves the way for a simple, quantitative and non-invasive method to measure skin biomechanical properties,” bridging a gap between subjective clinical exams and bulky lab equipment.

These works resonate with the broader technological trends that are redefining the limits of physiological sensing. Advances in ultra-low-power neural/biopotential front-ends are making long-term, high-fidelity electrophysiological recording feasible in mobile or implantable form factors [47,48]. Flexible and stretchable wearable bioelectronics based on soft conductors and nanocomposites are improving skin–sensor contact and comfort, which is essential for EEG, EMG, and long-wear physiological patches [49,50]. At the communication layer, optical and near-infrared links are being explored as high-bandwidth alternatives to RF for implantable and wearable medical devices, especially when data rates or tissue losses become limiting [51,52]. In parallel, recent advances in bio-microsystem integration and Lab-on-PCB technologies demonstrate how sensing, actuation, thermal control, and signal conditioning can be co-integrated within compact, manufacturable platforms, enabling lab-on-PCB and paper-/PCB-based LAMP systems that bring nucleic-acid assays into low-cost, portable hardware suitable for point-of-care or even body-proximal diagnostics [53,54,55,56]. Finally, bio-inspired polarization imaging shows how borrowing from animal visual systems can extend sensing beyond classical physiology toward navigation and contrast-enhanced biomedical imaging [57,58]. Collectively, these trends show that progress in circuits, materials, optics, and microsystems is expanding both the sensitivity and the situational reach of (bio)sensors, pushing the field toward multifunctional, networked, and truly wearable/implantable physiological monitoring.

## 3. Conclusions and Outlook

**Figure 7:**
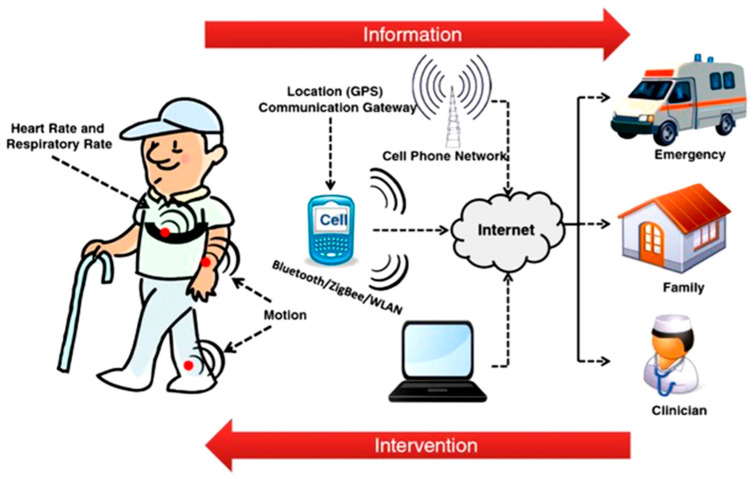
Conceptual overview and outlook of modern biosensing research.

Collectively, the contributions in the Special Issue illustrate the diversity and ambition in modern biosensing research. From wearable electrodes and amplifiers that improve how we capture signals to AI algorithms that enhance how we interpret them and integrated systems that combine multiple sensor modalities, the field is rapidly evolving on multiple fronts. A recurring theme is the drive towards more personalized real-time health monitoring, whether it is detecting an impending fall in older adult, a drop in a diver’s cognitive function, a spike in an athlete’s glucose, or subtle changes in a patient’s brain activity. The synergy of advanced sensors with machine learning is enabling the detection of patterns that were previously hidden in noisy data, allowing for earlier warnings and more proactive interventions. Another clear trend is interdisciplinarity: progress often comes from crossing traditional boundaries, for example, applying photonics to solve bio-data transmission limits or using techniques from social psychology with hyperscanning to study brain-to-brain interactions. By embracing such cross-cutting approaches, researchers are creating solutions that integrate physical, chemical, and biological sensing towards a more holistic understanding of human health. The interplay between sensor technologies, data interpretation, and interdisciplinary integration emerging from these works is schematically summarized in Figure 7.

Looking ahead, the works in the Special Issue point to several exciting directions. We can expect to see further miniaturization and power reduction in biosensor hardware, enabling large-scale sensor networks on (or in) the body without being a burden to the user. Similarly, the use of multi-modal data fusion and explainable AI will likely become the standard in physiological monitoring, as exemplified by several studies in the Special Issue. This will improve accuracy, but also user trust in technologies like health wearables and BCIs. The emphasis on non-invasive and comfortable monitoring is also evident: whether it is dry nanocomposite electrodes, optical glucose sensing, or nonintrusive stiffness assessment, the goal is to make monitoring seamless and pain-free so that data can be gathered continuously in everyday life rather than only in clinical settings. Finally, as these technologies mature, standardization and validation in real-world conditions will be crucial. Encouragingly, some authors in the Special Issue have already raised the call for community standards (e.g., for evaluating new EEG sensors), which will help accelerate translation of research prototypes into reliable products. In summary, the (bio)sensor field is poised to transform healthcare by pushing the frontiers of how, where, and what we can monitor. The rich array of developments showcased in this Special Issue, from AI-powered algorithms to novel device engineering, underscores a vibrant interdisciplinary effort to break down the boundaries of sensors, making the invisible measurable for the benefit of global health.

## Data Availability

No new data were created or analyzed in this study. Data sharing is not applicable to this article.

## Abbreviations

The following abbreviations are used in this manuscript:

AEP	Auditory Evoked Potential
AI	Artificial Intelligence
ASIC	Application-Specific Integrated Circuit
BCI	Brain–Computer Interface
CGM	Continuous Glucose Monitor/Continuous Glucose Monitoring
CNN	Convolutional Neural Network
DL	Deep Learning
ECG	Electrocardiography
EDA	Electrodermal Activity
EEG	Electroencephalography
EMG	Electromyography
fNIRS	Functional Near-Infrared Spectroscopy
HFnu	High-Frequency Power (normalized units)
HRV	Heart Rate Variability
IBAM	Impact-Based Analysis Method
IMU	Inertial Measurement Unit
LAMP	Loop-Mediated Isothermal Amplification
LBP	Low Back Pain
LSTM	Long Short-Term Memory (network)
MARD	Mean Absolute Relative Difference
ML	Machine Learning
Multi-AdaFNN	Multi-Adaptive Functional Neural Network
OGTT	Oral Glucose Tolerance Test
OWCS	Optical Wireless Communication System
PCB	Printed Circuit Board
PEF	Power Efficiency Factor
PID	Proportional–Integral–Derivative (controller)
PPG	Photoplethysmography
RF	Radio Frequency
RMSSD	Root Mean Square of Successive Differences
ROM	Range of Motion
rPPG	Remote Photoplethysmography
SNR	Signal-to-Noise Ratio
